# CD147慢病毒表达载体的构建及稳定转染A549细胞系的建立

**DOI:** 10.3779/j.issn.1009-3419.2012.12.03

**Published:** 2012-12-20

**Authors:** 绍兴 杨, 传昊 汤, 思涵 王, 三泰 宋, 晓晴 刘

**Affiliations:** 1 100071 北京，军事医学科学院附属医院肺部肿瘤科 Department of Pulmonary Oncology, Affiliated Hospital of Academy of Military Medical Sciences, Beijing 100071, China; 2 100071 北京，军事医学科学院附属医院乳腺肿瘤科 Department of Breast Oncology, Affiliated Hospital of Academy of Military Medical Sciences, Beijing 100071, China

**Keywords:** CD147, 慢病毒载体, 转染, 增殖, CD147, Lentiviral expression vector, Transfection, Proliferation

## Abstract

**背景与目的:**

CD147是一类位于肿瘤细胞膜表面的跨膜糖蛋白，可促进肿瘤的浸润和转移。本研究拟构建CD147慢病毒表达载体，建立稳定过表达*CD147*的人肺腺癌A549细胞系，观察过表达CD147后对MMP-9及细胞增殖、侵袭能力的影响。

**方法:**

RT-PCR扩增*CD147*基因全长序列，将序列插入pEGFP载体，构建pEGFP-CD147慢病毒表达载体，随后转入293FT细胞中进行慢病毒包装，用获得的慢病毒毒液感染人肺腺癌细胞系A549，建立稳定过表达CD147的A549细胞系。Real-time PCR检测MMP-9的变化情况，CCK-8及Transwell法检测人肺腺癌细胞增殖、侵袭能力的变化。

**结果:**

经限制性内切酶鉴定及测序分析，成功构建了pEGFP-CD147慢病毒表达载体质粒。Real-time PCR和Western blot检测显示，与对照组相比，转染pEGFP-CD147慢病毒表达载体组的细胞，CD147的表达在mRNA和蛋白两个水平均增高，成功建立了A549-CD147细胞系。上调CD147的表达后，MMP-9的mRNA表达水平明显升高。同时，A549-CD147细胞增殖和侵袭能力明显增加（*P* < 0.05）。

**结论:**

成功构建CD147慢病毒表达载体和A549-CD147细胞系，过表达CD147可上调MMP-9的表达，增强人肺腺癌细胞的增殖和侵袭能力。

## Abstract (en)

**Background and objective:**

CD147, a type of transmembrane glycoprotein embedded on the surface of tumor cells, can promote tumor invasion and metastasis. This aim of this study is to construct a CD147 lentiviral expression vector, establish its stably transfected A549 cell line, and observe the effect of CD147 on MMP-9 proliferation as well as on the invasive ability of human lung adenocarcinoma cells.

**Methods:**

Full-length *CD147* gene was amplified by real-time polymerase chain reaction (RT-PCR), inserted into a pEGFP vector to construct pEGFP-CD147 and pEGFP vectors, and then transfected into 293FT cells to precede the lentivirus equipment package. Subsequently, we collected the lentivirus venom to infect the A549 cells and establish a stable, overexpressed cell line named A549-CD147. The mRNA expression of MMP-9 was examined by RT-PCR. The proliferation and invasive ability of the human lung cancer cells before and after transfection were examined by the CCK-8 and Transwell methods.

**Results:**

A CD147 lentiviral expression vector (pEGFP-CD147) was successfully constructed by restrictive enzyme digestion and plasmid sequencing. RT-PCR and Western blot analyses revealed increased mRNA and protein expression of *CD147* gene in cells transfected with pEGFP-CD147 compared with the control groups. Therefore, the A549-CD147 cell line was successfully established through the experiment. The mRNA expression of MMP-9 also significantly increased after the upregulation of CD147 expression. Meanwhile, CCK-8 and Transwell assays indicated that the proliferation and invasive ability significantly increased in the A549-CD147 cells.

**Conclusion:**

A lentiviral CD147 expression vector and its A549 cell line (A549-CD14) were successfully constructed. CD147 overexpression upregulated the protein expression of MMP-9, and strengthened the proliferation and invasive ability of human lung adenocarcinoma cells.

肺癌是发病率和病死率均居第一位的肿瘤，其中非小细胞肺癌占80%，按其病理分型依次递减为腺癌、鳞癌、大细胞肺癌等。腺癌易发生血行转移，近年的研究^[1]^又发现其所占比例有所增长，成为女性和年轻患者中最为常见的一种类型，对肺腺癌细胞的生物学特性进行深入的研究有着非常重要的意义。CD147，又称基质金属蛋白酶诱导因子（EMMPRIN），是一类位于肿瘤细胞膜表面的跨膜糖蛋白，属于免疫球蛋白超家族，能够刺激成纤维细胞产生大量基质金属蛋白酶（matrix metalloproteinases, MMPs）^[2]^，可介导基底膜组成成分和细胞外基质大分子的重塑，促进肿瘤的浸润和转移，诱导肿瘤血管的生成^[3]^。尽管CD147的功能和作用在其它类型肿瘤研究中已有报道，但在肺腺癌细胞生物学特性中还未见研究报道。本实验拟通过构建CD147的慢病毒表达载体，建立稳定表达CD147的人肺腺癌A549细胞系，检测上调表达CD147后MMP-9及人肺腺癌细胞增殖、侵袭能力的变化情况，为研究CD147在人肺腺癌生物学特性中的功能和作用奠定基础。

## 1 材料和方法

### 1.1 材料

过表达慢病毒载体pEGFP购自Addgene公司，慢病毒包装质粒pLP1、pLP2、pLP/VSVG购自Invitrogen公司。pMD-T载体购自TaKaRa公司。SYBR Green real-time PCR mixture购自Qiagen公司。限制性内切酶、dNTP、Taq酶以及快速连接试剂盒均购自TaKaRa公司，引物合成及测序由上海英骏公司完成。CCK-8试剂盒购自DojinDo公司。转染试剂Lipofectamine 2000购自Invitrogen公司。慢病毒包装细胞293FT细胞购自Invitrogen公司。人肺腺癌细胞系A549由清华大学生命科学院肿瘤分子研究室惠赠。

### 1.2 方法

#### 1.2.1 引物设计

根据PubMed上*CD147*基因全长序列（NM 001728.3），以及pEGFP载体的酶切位点，进行引物设计：CD147 sense，5’ GCTAGCATCATGGCGGCTGCGCTGTT 3’，CD147 antisense，5’ TCTAGAGGAAGAGTTCCTCTGGCGGACGTT 3’。其中，分别在上游和下游的5’端加入了*Xba*I和*Nhe*I酶切位点。

#### 1.2.2 RT-PCR扩增人*CD147*全基因序列

用Trizol试剂按照说明书方法提取A549细胞总mRNA，然后取1 μg反转录成cDNA，用上述引物进行PCR扩增CD147全长序列，扩增条件如下：先94 ℃预变性5 min，94 ℃变性30 s，57 ℃退火30 s，72 ℃延伸90 s，共计30个循环反应，最后72 ℃延伸4 min，4 ℃保存。反应产物进行琼脂糖凝胶电泳，对所需片段进行凝胶回收，获得带有*Xba*I和*Nhe*I双酶切位点的CD147全基因组。

#### 1.2.3 人*CD147*基因的慢病毒过表达载体的构建及鉴定

将获得的CD147全基因PCR克隆到pMD-T载体中，转化大肠杆菌，进行蓝白斑筛选，挑取阳性克隆后在液体LB中扩大化培养，提取质粒后进行PCR及酶切鉴定，鉴定正确后送去测序。将测序验证后的重组质粒和pEGFP载体分别用*Xba*I和*Nhe*I进行双酶切，回收所需要的基因及载体片段，用快速连接试剂盒进行连接反应，16 ℃过夜连接后转化大肠杆菌，挑取阳性克隆后在液体LB中扩大化培养，提取质粒后进行PCR及酶切鉴定，鉴定正确后送去测序。将鉴定正确的CD147过表达载体命名为pEGFP-CD147。

#### 1.2.4 慢病毒的包装

慢病毒包装细胞293FT培养条件为添加10%FBS的H-DMEM，取5 μg pEGFP-CD147质粒及对照质粒pEGFP与包装质粒4.2 μg pLP1、2 μg pLP2和包膜质粒2.8 μg pLP/VSVG质粒在无血清培养基中与42 μL Lipofectamine 2000混合，室温孵育20 min，形成DNA-Lipofectamine 2000复合物后，转染293FT细胞，6 h后更换为含有1 mmol/L丙酮酸钠的培养基，48 h后收集培养基上清，用0.45 μM滤器过滤后，超速离心浓缩病毒，分装后-80 ℃保存。

#### 1.2.5 过表达CD147的A549细胞系的建立

人肺腺癌细胞系A549培养条件为添加10%FBS的H-DMEM，当A549细胞生长至60%密度时，吸去培养皿中的培养基，加入pEGFP-CD147和pEGFP慢病毒毒液，同时加入Polybrene，使其终浓度为8 mg/L，置37 ℃、5%CO_2_的孵箱中培养过夜。第2天，去除培养基，添加完全培养液，待细胞密度生长至80%-90%时，按1:3传代。流式细胞术分选表达绿色荧光蛋白的细胞。分别命名为A549-CD147、A549-pEGFP。

#### 1.2.6 细胞总mRNA的提取、逆转录反应及实时定量PCR检验干涉效率

用Trizol试剂溶解细胞，然后按照说明书方法提取细胞总mRNA，然后取1 μg反转录成cDNA，用SYBR GREEN mixture做染料，用iQ^TM^5多重实时荧光定量PCR仪进行实时定量PCR检测CD147的过表达效率，用GAPDH做内参，所用引物序列为：5′-CD147 sense，GCTAGCATCATGGCGGCTGCGCTGTT-3′，5′-CD147 antisense，5′-TCTAGAGGAAGAGTTCCTCTGGCGGACGTT-3′；GAPDH sense，5′-GAGTCAACGGATTTGGTCGT-3′，GAPDH antisense，5′-TTGATTTTGGAGGGATCTCG-3′；MMP-9正义链：5′-TGACAGCGACAAGAAGTG-3′，MMP9反义链：5′-CAGTGAAGCGGTACATAGG-3′。

#### 1.2.7 Western blot

用RIPA溶解细胞后提取细胞蛋白，各取200 μg进行SDS-PAGE，电泳后用电转仪进行转膜，5%脱脂奶粉室温封闭1 h后加入mouse anti-CD147抗体（1:300），4 ℃过夜，TBST洗涤3次，每次10 min，加入HRP偶联山羊抗小鼠二抗（1:1, 000），室温孵育1 h，TBST洗涤3次，每次15 min，ECL显影。同时以β-actin为内参照。

#### 1.2.8 CCK-8法检测细胞增殖能力

将A549细胞、A549-pEGFP、A549-CD147按照每孔1×10^4^/200 μL的密度接种于96孔板中继续培养，每组设置4个复孔，分别选取24 h、48 h、72 h三个时间节点进行检测。检测前换液1次，每孔加100 μL培养基和10 μL CCK-8，细胞放入37 ℃、5%CO_2_培养箱中培养2 h，使用酶联仪在450 nm波长检测光密度值。实验重复3次，取其平均值。

#### 1.2.9 细胞侵袭能力检测

使用Transwell（Corning Inc., USA）检测转染CD147组及对照组细胞的侵袭能力变化。首先，将Matrigel置于4 ℃过夜使其融化，用预冷的无血清1640以1:3稀释Matrigel，每个上室加入稀释后的Matrigel 100 μL，室温2 h使其凝固。然后于转染后24 h，收集A549-CD147、A549-pEGFP和A549细胞，用无血清1640液重悬细胞，细胞记数并调整细胞浓度为5×10^5^个/mL。每个上室中加入100 μL细胞悬液，下室加入含10%小牛血清的1640培养液，每种细胞做3孔。继续培养48 h后，取出滤膜，甲醇固定，常规HE染色。显微镜下计数迁移至滤膜外表面的细胞数，每张滤膜随机计数10个视野（×200），取平均值，实验重复3次。

### 1.3 统计学分析

使用SASS 9.1统计学软件进行数据处理，单组间的比较，采用*t*检验。多组间采用具有一个重复测量的两因素设计定量资料的方差分析，*P* < 0.05为差异具有统计学意义。

## 2 结果

### 2.1 pEGFP-CD147慢病毒表达载体的构建及鉴定

利用特异性引物扩增出了大小约为1, 150 bp的目的基因条带，与预期的大小片段一致（图 1A）。将测序验证正确的pMD-T-CD147进行双酶切，回收目的片段，并连接至pEGFP表达载体中，构建重组质粒pEGFP-CD147。提取质粒后进行*Xba*I和*Nhe*I双酶切，结果显示，酶切后呈现两条条带，与CD147目的基因片段和pEGFP载体片段的大小一致（图 1B），对质粒进行PCR，琼脂糖凝胶电泳后出现了与CD147大小一致的片段（图 1C），将酶切及PCR鉴定正确的质粒送去测序。测序的结果与预期的*CD147*基因序列完全相同。将鉴定正确的CD147过表达载体命名为pEGFP-CD147。

**1:**
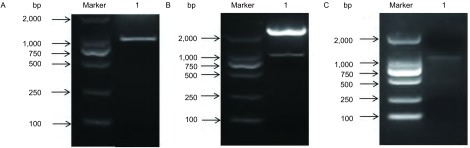
CD147慢病毒表达载体的构建及鉴定 Construction and identification of *CD147* lentiviral expression vector. A: Human *CD147* whole genome obtained; 1: Human *CD147* gene. B: Restriction map analysis of CD147 lentiviral expression vector; 1: pEGFP vector and *CD147* gene; C: Identification of CD147 lentiviral expression vector by RT-PCR; 1: *CD147* gene.

### 2.2 pEGFP-CD147慢病毒表达载体的包装和转染人肺腺癌A549细胞系

分别将pEGFP-CD147慢病毒表达载体和pEGFP空载体，与包装质粒pLP1、pLP2及包膜质粒pLP/VSVG、Lipofectamine 2000共转染293FT细胞，48 h后荧光显微镜下发现大部分细胞呈绿色荧光表达（图 2A）。收集病毒并去除漂浮细胞和细胞碎片，浓缩后分别感染A549细胞，48 h后荧光显微镜下均见到部分A549细胞发绿色荧光，说明pEGFP-CD147及pEGFP成功转入A549细胞中（图 2B）。连续将细胞培养1个月，分别提取感染pEGFP-CD147慢病毒表达载体的细胞和pEGFP-CD147空载体的细胞的总RNA。半定量RT-PCR分析表明，转染pEGFP-CD147慢病毒表达载体后的细胞，CD147的mRNA表达水平明显增高（图 2C），进一步的实时荧光定量PCR结果表明，对比转染pEGFP空载体组，转染pEGFP-CD147慢病毒表达载体后的细胞的CD147表达水平增加了2.5倍（图 2D）。说明所构建的载体在A549细胞中表达。

**2:**
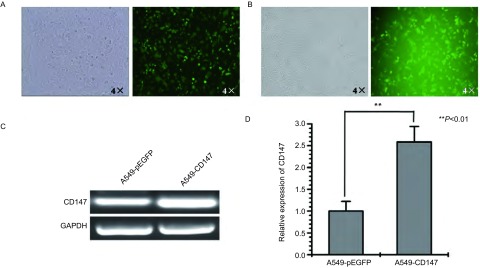
pEGFP-CD147慢病毒的包装和感染人肺腺癌A549细胞系 pEGFP-CD147 lentiviral packaging and infection of human lung adenocarcinoma cell line A549. A: pEGFP-CD147 lentiviral expression vector was transfected in 293FT cells; B: pEGFP-CD147 lentiviral venom infected A549 cells; C: Detection of CD147 mRNA expression levels in the respective cells by RT-PCR; D: Detection of CD147 mRNA expression levels in the respective cells by real-time PCR.

### 2.3 A549-CD147细胞系的建立

选取转染pEGFP-CD147慢病毒表达载体的A549细胞和转染pEGFP空载体的A549细胞进行流式细胞术分选（图 3A），分选后经扩增培养1个月，荧光显微镜下可见满视野的绿色荧光标记的细胞（图 3B）。两组细胞的real-time PCR分析显示，转染pEGFP-CD147组细胞的CD147的mRNA表达水平比转染pEGFP空载体组高17.32倍（图 3C），Western blot结果表明，与对照组相比，pEGFP-CD147组细胞的CD147蛋白表达水平明显升高（图 3D），表明我们已经成功建立了稳定过表达CD147的人肺腺癌A549细胞系，命名为A549-CD147。

**3:**
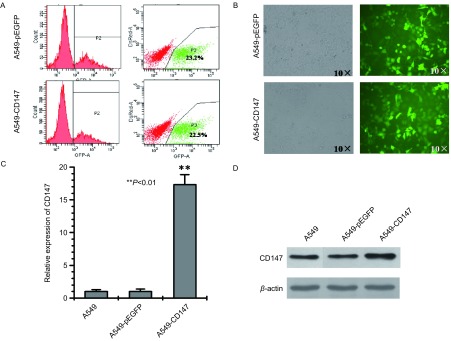
A549-CD147细胞系的建立 Established the A549-CD147 cell line. A: The A549 cells transfected with p-EGFP lentiviral expression vector and p-EGFP empty vector were sorted by flow cytometry respectively; B: The A549-pEGFP cells and A549-CD147 cells after sorted by flow cytometry; C: Detection of CD147 mRNA expression levels in the respective cells by real-time PCR; D: Detection of CD147 in protein level by Western blot in cells.

### 2.4 过表达CD147对MMP-9及人肺癌细胞增殖、侵袭能力的影响

Real-time PCR结果显示转染*CD147*基因后，A549-CD147细胞中MMP-9 mRNA的表达明显增加（*P* < 0.001）。CCK-8法检测结果表明，转染后的第1天，3种细胞的增殖率没有明显差别，而第2、3天，与对照组相比，A549-CD147细胞的增殖能力明显增强。A549-pEGFP细胞与A549细胞的增殖能力对比未见统计学差异（*P* < 0.001）（图 4）。Transwell结果显示，穿透Matrigel到达滤膜的A549-CD147细胞数明显多于A549-pEGFP和A549细胞（*P* < 0.001）。

**4:**
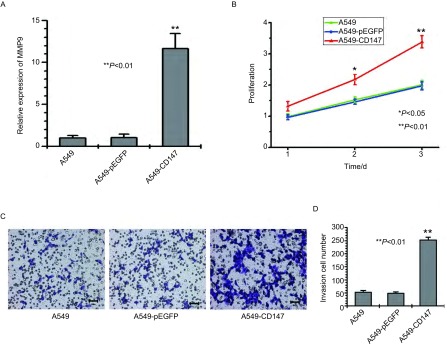
过表达CD147对MMP-9及人肺癌细胞增殖、侵袭能力的影响 Effects on MMP-9, proliferation and invasive ability of the human lung adenocarcinoma cells after overexpression CD147. A: The relative expression rate of MMP-9 mRNA was significantly enhanced in A549-CD147 cells than that in A549 and A549-pEGFP cells; B: The growth curves indicated that the growth rates had not significant difference among A549-CD147, A549-pEGFP and A549 cells in the first day after transfection, but in the second day (*P* < 0.05) and the third day (*P* < 0.01), the growth rate was significantly higher in A549-CD147 cells than that in A549-pEGFP and A549 cells; C: Representative microscope field of filters under the Matrigel from A549-CD147, A549-pEGFP and A549 cells respectively (×100); D: The Histogram showed that the number of invasive cell was significantly more in A549-CD147 cells than that in A549-pEGFP and A549 cells (*P* < 0.01).

## 3 讨论

在包括对卵巢、肺、前列腺、胰腺、乳腺等恶性肿瘤的研究中发现，MMPS的活性程度与肿瘤的侵袭转移潜能关系密切。而肿瘤细胞表面高表达的CD147使得MMPS表达数量及活性增加，从而降解基底膜的主要成分，破坏天然组织的机械屏障，促进肿瘤的浸润和转移^[4]^。CD147是一个在多种生理和病理过程中发挥重要作用的关键分子，但大家关注的重点还是其在肿瘤生长和发展中的功能和作用。Zheng等^[5]^发现上调CD147的表达可促进胃癌细胞的生长和血管的生成。Wang等^[6]^在体外实验研究中发现，CD147的表达与胃癌SGC7901细胞的增殖速度密切相关。Bougatef等^[7]^发现CD147的表达可促进恶性黑色素细胞瘤细胞的浸润、转移。Hao等^[8]^已证实CD147的表达可能是前列腺癌细胞耐药的一个关键因素。为探讨CD147在肺腺癌细胞生物学特性中的功能和作用，我们首先构建了CD147慢病毒表达载体和稳定表达CD147的人肺腺癌A549细胞系。

对比其它研究应用的普通质粒载体的方法，我们所构建的慢病毒载体具有获得病毒周期短，滴度高，可以感染分裂、非分裂细胞，能将外源基因高效导入宿主细胞，从而在细胞中稳定长期表达siRNA、cDNA或报告基因，且不会产生化学转染或腺病毒转染引起的细胞损伤及免疫反应。慢病毒实验系统被广泛应用于各类基因治疗的实验研究中^[9, 10]^。为实现*CD147*基因在人肺腺癌A549细胞中的高效、稳定表达，我们首先成功构建了pEGFP-CD147重组表达质粒，随后进行293FT细胞包装慢病毒。因我们采用的慢病毒载体上表达GFP，慢病毒毒液感染A549细胞后，经荧光显微镜观察可以见到部分细胞中有GFP表达，表明A549细胞已经有效地被慢病毒感染。感染后的细胞经过流式细胞术分选，培养扩增1个月后，细胞荧光显示镜下可见到满视野的绿色荧光标记的细胞。Real-time PCR结果显示，与对照组相比，pEGFP-CD147组CD147的表达水平上调17.29倍，Western blot检测结果也显示，pEGFP-CD147组的CD147的表达在蛋白水平也明显增加。说明我们已经成功建立了过表达CD147的人肺腺癌A549细胞系，命名为A549-CD147。我们随后对CD147靶产物MMPS家族的MMP-9的表达水平进行了real-time PCR检测，发现过表达CD147可上调MMP-9的表达。CCK-8和Transwell法证实A549-CD147细胞的增殖和侵袭能力明显增强。根据上述研究所显示CD147具有的功能特性，推测其有可能是一个潜在的治疗靶点，这也是我们后续研究关注的重点。

综上所述，我们成功构建了CD147慢病毒表达载体，建立了稳定上调表达CD147的A549-CD147细胞系，并对其在促进细胞增殖和侵袭方面进行了初步的研究。我们将利用成功构建载体和细胞系进一步研究CD147在人肺腺癌生物学特性中的功能和作用。
